# In-vitro reconfigurability of native chemical automata, the inclusiveness of their hierarchy and their thermodynamics

**DOI:** 10.1038/s41598-020-63576-6

**Published:** 2020-04-22

**Authors:** Marta Dueñas-Díez, Juan Pérez-Mercader

**Affiliations:** 1 Department of Earth and Planetary Sciences and Origins of Life Initiative, Harvard University, Cambridge, Massachusetts, 02138-1204 United States; 2 Repsol Technology Lab, c/ Agustín de Betancourt, s/n., 28935 Móstoles Madrid, Spain; 3 Santa Fe Institute, Santa Fe, New Mexico 87501 United States

**Keywords:** Computer science, Physical chemistry

## Abstract

Living systems process information using chemistry. Computations can be viewed as language recognition problems where both languages and automata recognizing them form an inclusive hierarchy. Chemical realizations, without using biochemistry, of the main classes of computing automata, Finite Automata (FA), 1-stack Push Down Automata (1-PDA) and Turing Machine (TM) have recently been presented. These use chemistry for the representation of input information, its processing and output information. The Turing machine uses the Belousov-Zhabotinsky (BZ) oscillatory reaction to recognize a representative Context-Sensitive Language (CSL), the 1-PDA uses a pH network to recognize a Context Free Language (CFL) and a FA for a Regular Language (RL) uses a precipitation reaction. By chemically reconfiguring them to recognize representative languages in the lower classes of the Chomsky hierarchy we illustrate the inclusiveness of the hierarchy of native chemical automata. These examples open the door for chemical programming without biochemistry. Furthermore, the thermodynamic metric originally introduced to identify the accept/reject state of the chemical output for the CSL, can equally be used for recognizing CFL and RL by the automata. Finally, we point out how the chemical and thermodynamic duality of accept/reject criteria can be used in the optimization of the energetics and efficiency of computations.

## Introduction

Chemical reactions are the ultimate recognition machines: molecules of the reacting substances meet in space-time and “recognize”, to then combine and transform into different substances, the reaction products. This transformation contains all the elements for what is a very high level (heuristic) definition of a computation: information is input, mechanically (i.e., systematically) transformed and output in some useful form. The process involves energy and information transfer and comes accompanied by changes in the state functions of the chemical system.

Given some environmental conditions, the same concentrations of reactants fed to the reactor in the same order (cf. below) will lead to the same products and quantities. That is, the chemical reaction responds mechanically (i.e. repetitively) to its information carrying chemical inputs and therefore can, in principle, be thought of as an automaton.

The reaction will take place under some conditions if the interacting molecules have the appropriate electronic configurations to recognize themselves (react) and if they are fed in the appropriate order and proportions (also called aliquots). Molecular geometries, electronic configurations and aliquots can be thought of as carriers of information. Both digital (i.e. discrete) and analog (i.e. continuous) information components play a role in the chemical reaction. The products of the reaction are therefore the result of the transformation by the reaction automaton of the initial information contained in the aliquots of the reactants.

Chemical reactions occur via a series of fast intermediate (sub) reactions. The set of these constitute the reaction mechanism or reaction network. In this network the nodes (intermediate species) are connected by reaction pathways. What reaction pathway is actually followed depends not only on the reactants added to the reaction, but also on the order in which their aliquots are fed to the reactor^1^. In general, not all pathways are equally fast or slow and the frequency with which they are visited during the consumption of reactants and production of reaction products depends on the precise reaction vessel conditions, aliquot composition, as well as the order in which the aliquots are fed. (Note that for non-linear chemical kinetics, one expects that the order in which the aliquots are fed to the reaction be important, as non-linear mathematical operations are generally non-commutative; for example, x^y^ $\ne$ y^x^ or, log sin x $\ne$ sin log x. (This observation is particularly interesting in the light of the properties of a computation^2^.)

In summary, a chemical reaction can be thought of as an automaton which processes the information contained in an ordered sequence of aliquots. The generation of the reaction products can therefore be viewed as the transformation of the original information in the sequence of aliquots into some output information now residing in the products and the final physical conditions of the reactor. (And these can, in turn, carry out some work or perform a function.)

The above paragraph together with the mechanical (i.e. repetitive) nature of a chemical reaction allows one to think of the operation of a chemical reaction in full as a “computation carried out by chemistry”: information is input by a sequence of aliquots, mechanically processed by the chemical automaton and finally delivered in the form of the information contained in the final state of the reaction and its products. We call this “native chemical computation”. In other words, chemical reactions, which occur at the Angstrom scale and in a number of 6.023 ×10^23^ molecules per cubic centimeter, are the ultimate molecular recognition automata^1^. But can we harness this power? A step to take would be to make them programmable using the fact that the frequency and strength with which the various internal reaction pathways are visited depend on the details of the aliquot choice and on the order with which they are fed to the reaction. Thus, we can introduce a means to program the chemical automaton in order to recognize a variety of sequences of tokens (letters of an alphabet.)

As is well known abstract automata process information contained in sequences of tokens (letters in the alphabet) that belong to some language. These are classified in the inclusive four-level Chomsky language hierarchy^3^ from the simplest Regular Languages (RL) to the most complex, the Recursive Enumerable Languages. Correspondingly, the automata, which identify at least one language in its class and none above this class, are also arranged in an inclusive hierarchy^4^. That is, an automaton will recognize at least one language in its class and one or more languages in each of the lower levels in the hierarchy. Hence a chemical automaton should be able to process chemical information in the same way that abstract automata.

Since any computation can be carried out as a collection of interconnected word recognition (acceptance and rejection of words) problems^5^ it is then important to ask the question of whether chemical automata form an inclusive hierarchy.

In a recent paper^1,6^ we have demonstrated that chemical computation at any level of the Chomsky hierarchy does not require the intervention of biochemistry. We have built individual physico-chemical realizations^1,6,7^ of each of the automata in the Chomsky hierarchy without any biochemistry and without large carbon molecules. We have done this by showing (a) how the letters of an alphabet can be represented chemically, (b) how to represent words in languages at the various levels of the Chomsky hierarchy using sequences of these letters, (c) how by feeding sequences of these chemicals representing words in chemical reactions of various levels of complexity act as automata and (d) we have introduced a physico-chemical measure that characterizes the thermodynamics of chemical computation. That is, one can represent the (practical) levels of automata in the Chomsky hierarchy in terms of chemical reactions of various levels of complexity^8^. But one still needs to show that automata form an inclusive hierarchy.

For the important case of the Turing machine^9^ we showed that there exists a free-energy and reaction extent related thermodynamic metric^1,6^ which can be used to characterize the results from processing a sequence. The measure has dimensions of action and is related to the free-energy dissipated by the chemical reaction in the recognition of the sequence. We also saw that the chemical description of the alphabet symbols for the words in a language can be macroscopically adjusted so that all words in the language accepted by the Turing machine have the same value of this thermodynamic measure. This is fundamental, as it implies that the Turing machine can be programmed and play a fundamental role in problems that require pattern recognition.

In this paper we will prove experimentally that the chemical Chomsky hierarchy is an inclusive hierarchy, as in the case of the abstract automata. We will do this by showing that in complete parallel with their abstract counterparts, the chemical realizations of automata not only recognize languages at their level but also below in the Chomsky hierarchy. That is the hierarchy of chemical automata is also inclusive. We will also extend the use of the thermodynamic metric mentioned above and previously introduced for the Turing machine, to the automata below the Turing machine rank in the hierarchy. This then shows that the thermodynamic interpretation of a computation with chemical automata is universal, in the sense that there exists a thermodynamic interpretation applicable to all automata. Finally, we will do all the above without using any biochemistry, so that our conclusions imply that full computation with chemistry does not require biochemistry.

## Results

**Figure 1:**
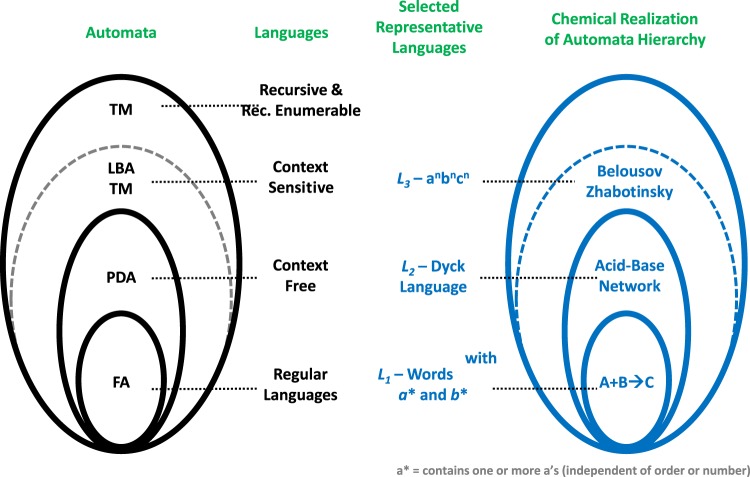
The classical automata hierarchy and the associated Chomsky hierarchy of languages. We show some representative languages and the chemical reaction systems used for their respective experimental implementation: (**a**) bimolecular elementary reaction for the language of all words containing at least one a and one b; a diprotic weak acid and strong base pH system for the Dyck language, and the Belousov-Zhabotinsky oscillatory chemistry for *L*_3_ = {*a*^*n*^*b*^*n*^*c*^*n*^, where *n* ≥ 1}. At the abstract level this hierarchy is inclusive. The work in this paper shows that the chemically realized automata also constitute an inclusive hierarchy.

We know^1,6^ that a native chemical Finite Automaton implemented by a simple precipitation reaction recognizes a Regular Language, *L*_1_ (see Fig. 1). Similarly, a native chemical 1-stack Pushdown automaton based on pH chemistry was shown to recognize the Dyck language, a Context-Free Language *L*_2_. We also designed a native Turing machine based on Belousov-Zhabotinsky chemistry capable of recognizing a well-known context sensitive language, *L*_3_ = {*a*^*n*^*b*^*n*^*c*^*n*^, where *n* ≥ 1} (see Fig. 1). We have already demonstrated that BZ can recognize the Dyck language using the formalism of multi-tape Turing Machines^7^. In what follows we will use these three languages as reference languages to characterize recognizing automata at the appropriate levels in the Chomsky hierarchy of languages^3^. (Since these are actual material implementations of the automata, their tapes cannot be infinite (or unbounded) as in theoretical implementations. By a series of strategies (cf. caption to Fig. 1 in ref. 1) one can increase the tape length. As noted by Minsky in ref. ^21^, an infinite tape is impossible in practice, since it would require infinite energy to be implemented.)

### Abstract automata recognition of the Dyck language, a context-free language

We choose the Dyck language, a context free language in the Chomsky hierarchy, to test the inclusiveness of native chemical automata. The Dyck language is a classic example in automata theory of a context free language. Indeed, abstract 1-stack PDA and Turing Machine descriptions for recognizing the Dyck language are well-known and available^10,11^.

The Dyck language or language of balanced parentheses consists of all the strings of open and closed parentheses satisfying the following two rules: Rule-1 During processing of the string the number of “)” never exceeds the number of “(”. Rule-2 Once the string is fully processed there are as many open parentheses “(” as closed parentheses “)”. Different classes of abstract automata check systematically these two rules in different ways thus affecting the number of steps, state transitions and computation time of the automaton.

An abstract 1-stack PDA recognizes the Dyck language by a procedure that keeps track of the difference between the number of open parentheses and closed parentheses in a limited-access (last-in first-out) type of memory called a “stack”^10,11^. Whenever a “(” is read by the PDA, an element is added (“pushed”) to the top of the stack and whenever a closed parentheses “)” is read, an element is removed (“popped”) from the top of the stack^10,11^. If during computation the automaton attempts to pop from an empty stack, Rule-1 above is violated, the automaton rejects the string (reject R_1_) and halts. Once the string is processed in its entirety, i.e. the end-of-sequence symbol (‘#’) is read, and if the stack is nonempty, Rule-2 above is violated and the automaton rejects the string (reject R_2_). Otherwise, the string is accepted, i.e. it is in L_2_.

An abstract Turing Machine, in contrast to the 1-stack PDA, has access to a less limited memory, its tape, with unrestricted access because the head can move forward and backwards over the tape, and can also read and write symbols on the tape. A common Turing Machine implementation^10,11^ for recognizing the Dyck language is as follows: once the sequence is on the tape, the head is located at the first symbol and moves to the “right” looking for a closed parenthesis, marking it with a distinct symbol, e.g. X, and reversing the head direction (to the “left”) until it finds the closest matching open parenthesis, overwriting it with an X and reversing again the head direction. These rules are then repeated iteratively. If during computation, the head reaches the beginning of sequence symbol while moving left, the automaton rejects the string (reject R_1_) and halts. Otherwise, once all the input symbols are processed, i.e. the automaton reaches the end-of-sequence symbol, the head direction is reversed to do a final check. If an open parenthesis is encountered before reaching the beginning of the expression, the automaton rejects the string (reject R_2_). Otherwise, the string is accepted.

We note that although the computation is executed differently by the abstract 1-stack PDA and Turing Machine, the number and types of rejects are the same: R_1_ corresponds to out-of-order “)” and can occur any time during computation; and R_2_ corresponds to excess “(” which in both the 1-stack PDA and the Turing machine will reject the input at the end of computation. The accept state can only be reached at the end of computation, and *only* if Rule-1 and Rule-2 above are satisfied.

### Chemical recognition of the Dyck language by the BZ reaction

The Belousov-Zhabotinsky reaction^12–14^ was discovered by Belousov in the 1950s while looking for a chemical analog of the Krebs cycle^15,16^. This nonbiochemical oscillatory chemistry, consists of the oxidation of a weak organic acid in an acidic aqueous solution by bromate ions in the presence of a transition metal catalyst. (There are in the literature several variants of its kinetic mechanism^17–19^ that have been extensively studied). The features of the chemical relaxation oscillations (period, amplitude, etc) depend on the reactant and intermediate concentrations, and importantly, on the order in which they are added to the reaction, hence the capability of this reaction to carry out computations^1^.

In the design and implementation of native chemical automata, one must adequately choose the chemical species and aliquots intended to represent the alphabet symbols of the language to be recognized, accepted or rejected. Our criterion is that a chemical species can represent an alphabet symbol if it affects a uniquely distinct pathway in the reaction network which, in turn, translates into a distinct non-oscillatory or oscillatory signature^1,6^. Based on our previous results^1,7^ an appropriate assignment for the Dyck language alphabet is: “(”- an aliquot of the oxidizer, sodium bromate; “)” – an aliquot of the reductant, malonic acid; and “#” – an aliquot of the catalyst, in this case tris(2,2′-bipyridyl) dichloro ruthenium(II), although the system will work independently of the specific chemical nature of the BZ transition metal catalyst. (More details in Methods section.)

**Figure 2:**
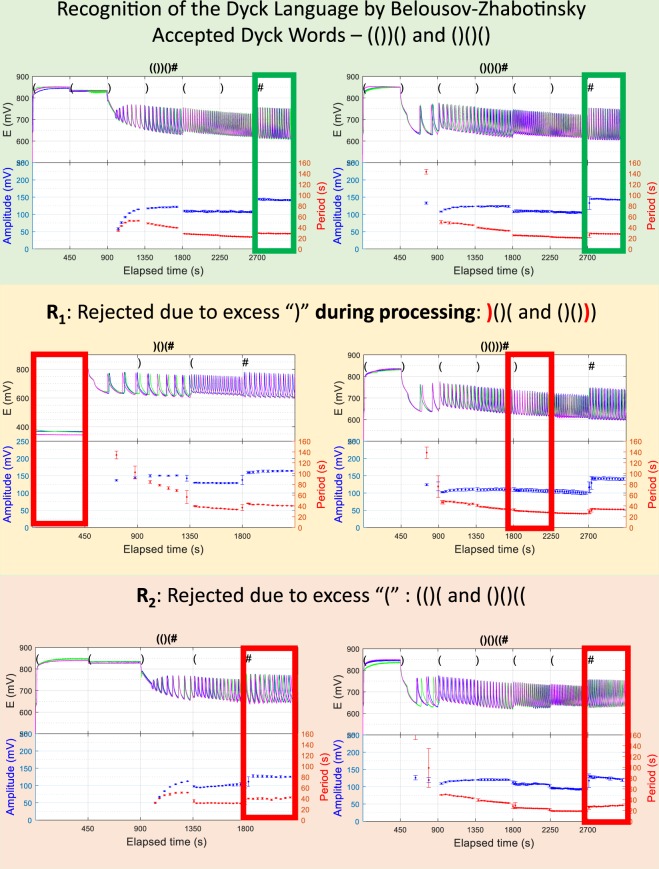
Recognition of the Dyck Language by Belousov-Zhabotinsky chemistry. Experimentally, each sequence was repeated 3 times, in which the period and amplitude were measured. The top panel shows the redox profiles for accepted words (())() and ()()(); the middle panel shows the sequences rejected due to excess closed parentheses during processing)()( and ()())), rejected on the first and fifth symbol respectively; and the bottom panel shows two sequences rejected at the end of computation since the number of open and closed parentheses is not the same (()( and ()()((, characterized by a too small final amplitude of oscillations.

**Figure 3:**
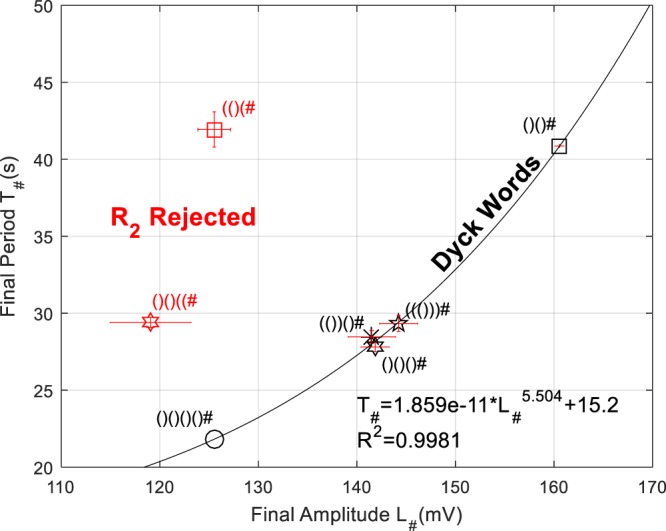
BZ-TM check of Rule-2: Equal number of open and closed parentheses. Accepted words lie on the nonlinear locus, showing a power law dependence of the final period *T*_#_ on the final amplitude *L*_#_. The plot shows 5 accepted sequences: ()(), ()()(), (())(), ((())), ()()()(). And 2 rejected sequences due to excess open parentheses, (()( and ()()((, contained in them.

The next, and critical, step is to identify the distinct oscillatory signatures associated with the reject and accept states. To check whether Rule-2 is satisfied or violated once the complete sequence has been processed, a chemical native TM uses two descriptors (cf. Fig. 2 for a representation of these features): one related to the frequency of the oscillations (here the period T is the time interval between two consecutive peaks) and the other related to the amplitude or location in the redox range of the oscillations (here the amplitude L is the difference between a peak value and its next trough value). Hence, if the final values of the pair [T, L] fall at the nonlinear locus [T_#_, L_#_] (cf. Fig. 3) the sequence is accepted. Otherwise, if the final oscillations have higher period and lower amplitude than the above locus, the automaton has rejected the sequence (R_2_-reject). Note also that an R_1_-reject (due to an excess of closed parentheses) is output by this chemical automaton as either a constant minimum redox potential if the system is not in an oscillatory regime yet, or as a smooth continuous period decrease (the derivative of the period with respect to time is differentiable) if the reaction is not yet in an oscillatory regime.

Figure 2 illustrates the experimental results. The top panel shows two examples of accepted strings ()()() and ((())), the middle panel shows two rejected strings due to excess “)” during computation for)()((and for ()())); and the bottom panel shows two rejected strings due to an unequal number of open and closed parentheses at the end of computation, for ()((and ()()((, respectively.

Figure 3 shows the [T_#_, L_#_] plot for the set of experimental sequences that would reach the end-of-sequence symbol (acceptance and R_2_- type rejection). The locus of accepted words gives (of course to be expected) a nonlinear power law dependence between T_#_ and L_#_. Rejected sequences due to excess “(” are displaced towards lower amplitudes and slightly higher periods.

In comparing the BZ-based TM and the pH-based 1-stack PDA used for the recognition of the Dyck Language, we note that the pH-based 1-stack PDA has the advantage of using a one-dimensional Rule-2 criterion (pH). However, it is also interesting to note that by using a thermodynamic-based signature, the Rule-2 checking criterion in the BZ-machine may be simplified into a one-dimensional criterion^1,6^ using the following area metric ${A}^{({Word})}$ introduced in refs. ^1,6^:

$${A}^{(Word)}={V}_{max}\cdot {t}_{interval}-{\int }_{{t}_{{\rm{\#}}}}^{{t}_{{\rm{\#}}}+{t}_{interval}}{V}_{osc}(t)dt\,\propto \,(\Delta {G}^{{\rm{{\prime} }}}\cdot {t}_{interval}-{\int }_{{t}_{{\rm{\#}}}}^{{t}_{{\rm{\#}}}+{t}_{interval}}\Delta {G}_{osc}(t)\cdot dt).$$

The *A*^(*Word*)^ metric measures how far from the maximum attainable Gibbs free-energy (i.e. when all catalyst is in its oxidized form) Δ*G*, is the Gibbs free-energy associated with the chemical oscillations output by the automaton once the full input string has been processed (this assumes that both terms are integrated over an equally long time interval). Remarkably, for the chemical automata-language pairings that result in a constant ${A}^{({Word})}$ independently of word length, the increase in the overall extent of reaction of the oxidation pathway as word length grows is compensated by an equivalent increase in the overall extent of reaction in the reduction pathway^6^.

**Figure 4:**
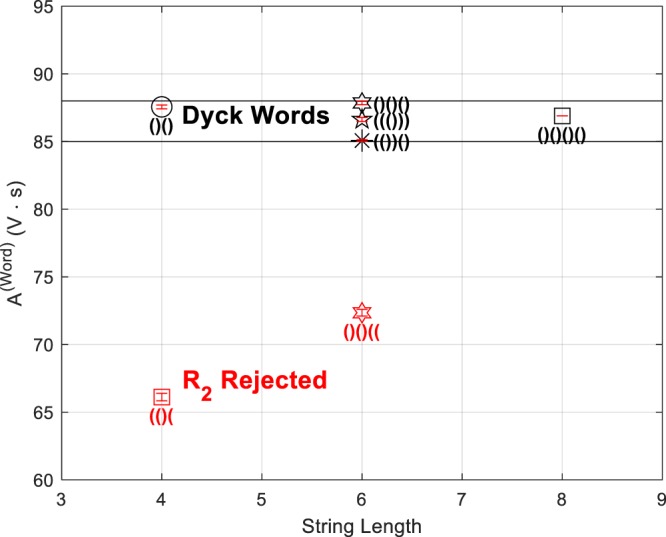
BZ-TM check of Rule-2 by thermodynamic signature. In this series of experiments, accepted words have an *A*^*(Word)*^ value of 86.5 ± 1.5, and sequences rejected due to excess open parentheses are displaced towards lower *A*^*(Word)*^ values. The plot shows 5 accepted words: ()(), ()()(), (())(), ((())), ()()()() and 2 rejected sequences due to excess open parentheses (()( and ()()((.

The Rule-2 checking criterion for this implementation (and of course given its chemical recipe) is as follows: accepted words have an *A*^(*Word*)^ value of 86.5 ± 1.5, while rejected sequences have a lower *A*^(*Word*)^ value (see Fig. 4).

### Recognition of L_1_ by Belousov-Zhabotinsky and the pH network

**Figure 5:**
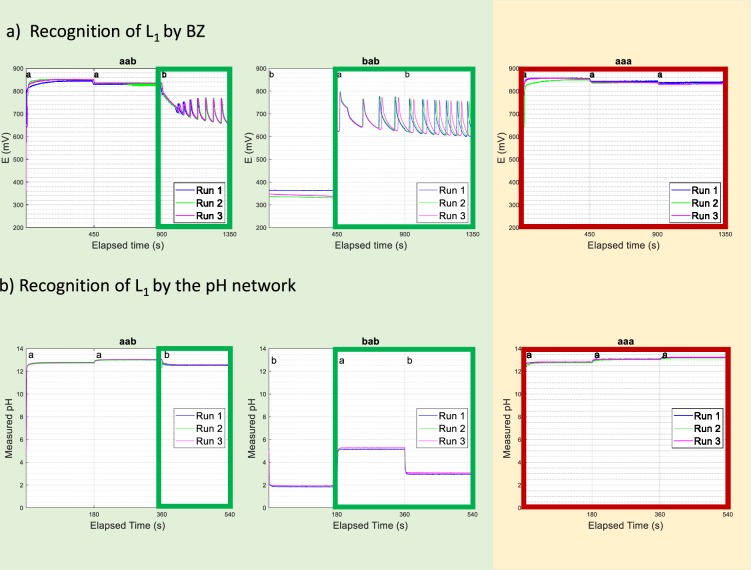
Recognition of L_1_ by both the Belousov-Zhabotinsky chemistry and by pH chemistry. Panel a) shows the experimental results of a BZ-TM recognizing L_1_: aab and bab are recognized (there is onset of oscillations) and aaa is rejected (flat high redox potential). Panel b) shows the experimental results of a pH-PDA recognizing L_1_: aab and bab are recognized as soon as a sign change in the pH step change is detected, and aaa is rejected since all pH step changes are ascending.

We denote the language of all words that contain at least one “a” and one “b” by L_1_. This is a regular language and can therefore be recognized by a finite automaton as well as by abstract automata higher in the hierarchy such as the 1-stack PDA and the Turing Machine. The experimental results for our chemical 1-stack PDA and TM are shown in Fig. 5.

A typical abstract finite automaton recognizing L_1_ has three distinct states: S_1_ is the active state so long as one or more “a” is read, S_2_ is the active state as long as one or more “b” is read, and S_3_ is the state reached when the symbol that had not yet been read is read (“b” if the previous symbols were all “a” or “a” if all the previous symbols were “b”). S_3_ is the only accept state and both S_1_ and S_2_ are reject states.

For the recognition of L_1_ by the BZ reaction we start by assigning sodium bromate to symbol “a” and malonic acid to “b”. The aliquot recipes of “a” and “b” can be kept the same as the recipes for “(” and “)”, respectively, in the above BZ implementation for recognition of the Dyck language. S_1_ is given by a flat high redox potential (catalyst dominantly in oxidized form), S_2_ by a flat low redox potential (catalyst dominantly in the reduced form) and, of course, S_3_ by the onset of oscillations. Figure 5 shows the experimental results of accepted words “aab” and “bab”, and the rejected string “aaa”.

The pH network already used to recognize the Dyck language, can also recognize L_1_ by assigning sodium hydroxide to symbol “a” and malonic acid to symbol “b”, and keeping the same aliquot recipes that were used to recognize L_2_ in ref. ^1^. In this chemical automaton, S_1_ is given by ascending pH step changes or maximum pH, S_2_ by descending pH step changes or minimum pH, and S_3_ by a change of sign in the pH step change (i.e. if the pH had been ascending and then decreases, or if the pH had been descending and then increases upon addition of a symbol).

## Discussion

We have successfully reconfigured a BZ-based Turing Machine such that in addition to the context sensitive language, L_3_, it can recognize the context-free language of balanced parentheses and/or the regular language L_1_. Similarly, we have shown how to reconfigure the pH-based 1 stack PDA to recognize L_1_. These results completely align our chemical automata with abstract automata theory and show that the chemical automata conform to a hierarchy that is inclusive, i.e. automata with higher complexity in the hierarchy can recognize languages at its level and also languages at lower complexity levels.

We can compare the pH-based 1-stack PDA and the BZ-based TM for recognizing the Dyck Language. The pH-based machine used a strong base, sodium hydroxide, as “(”, and a diprotic acid, malonic acid, as “)”, together with an appropriate pH indicator as “#”^1^. The aliquots were chosen so that one aliquot of “(” and one aliquot of “)” drive the reaction to the midpoint in pH after the first equivalence point, i.e. the point at which C_3_H_3_O_4_^−^ is in chemical equilibrium with C_3_H_2_O_4_^2−^.

The chemical signatures for the accept and reject states are compared in Table 1. The pH machine checks Rule-2 as follows: a pH at exactly the midpoint indicates accept, and a pH above the midpoint indicates excess open parentheses and vice versa (see below). The BZ-TM uses instead two descriptors (the period and amplitude of the oscillations) to carry out the final check on whether the number of open and closed parentheses is the same or not. Two descriptors are needed because an abstract TM is equivalent to a 2-stack PDA^20,21^, in which one stack simulates when the head moves to the right, and the other when the head moves to the left. Checking that [T_#_, L_#_] lies at the correct nonlinear T_#_ = *f*(L_#_) locus is equivalent to checking that the two stacks are empty. In the case of an excess of closed parentheses type of reject (R_1_), a distinct chemical signature (i.e. never occurring in accepted words) is needed: pH below midpoint provides such a distinct signature in the case of the pH-based PDA, while a smooth decrease of the period trend is the distinct signature in the case of the BZ-based automaton (as opposed to the observed discontinuous stepwise changes of period trends in accepted words).

**Table 1:** Comparison of the chemical signatures of accept and reject states for the pH-based 1-stack PDA and the BZ-based TM that recognize the Dyck Language.

	Accept	Type 1 Reject (R_1_)	Type 2 Reject (R_2_)
pH-based 1-stack PDA	pH_#_ = pH_midpoint	pH < pH_midpoint	pH_#_ > pH_midpoint
BZ-based TM	[T_#_, L_#_] at locus T_#_ = *f*(L_#_)	Continuous Period Decrease	[T_#_, L_#_] not at locus T_#_ = *f*(L_#_)

**Figure 6:**
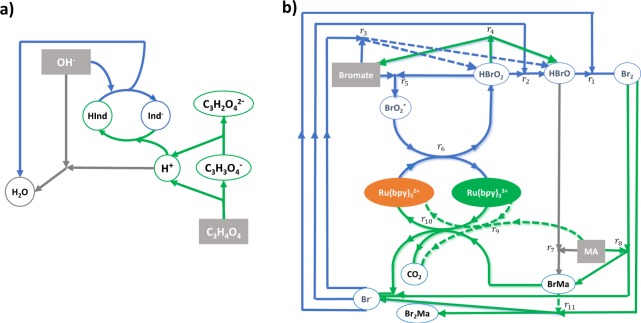
Reaction mechanisms for the pH-based 1-stack PDA and for the BZ-based TM. Panel a) shows the pH mechanism where “(” is NaOH, activating the “basic” pathway (blue arrows), “)” is the malonic acid, activating the “acid” pathway (green arrows), and the key intermediate is H^+^; Hind and Ind^−^ represent the protonated and deprotonated configurations of the pH indicator respectively. Panel b) shows a suitably modified version of the BZ-mechanism, “(” is sodium bromate activating the catalyst oxidation pathway; “)” is malonic acid activating the catalyst reduction pathway; and the key intermediates are Br^−^, HBrO_2_, HBrO, Br_2_, Bromomalonic acid, and H^+^. Arrows in blue consume protons, in green generate protons, and in grey neither consume nor generate protons.

The fact that a TM uses a more general and powerful memory than a one-stack PDA, translates in chemical terms into a more complex reaction network with a larger number of key intermediates and feasible chemical pathways. Figure 6 compares the reaction network of the pH-based 1-stack PDA with the BZ-based TM. The key intermediate in the pH network is the proton concentration H^+^, while BZ has several key intermediates, such as the bromide ion Br^−^ and the bromous acid HBrO_2_, but bromine Br_2_, hypobromous acid HBrO, proton H^+^ and bromomalonic acid are also critical intermediates for performing native computations. On the other hand, the pH network has two dominant pathways: the “acid” pathway (green arrows in Fig. 6 Panel a) and the “basic” pathway (blue arrows in Fig. 6 Panel a). Of course, the BZ network has a larger number of dominant pathways and four^1^ were clearly identified in the recognition of L_3_^1^. It is also interesting to note that the BZ reaction network can be easily coupled to other chemistries, paving the way to increase the number of feasible pathways in the coupled network. This can be done by exploiting the high reactivity of BZ with many chemical species, including organic compounds, inorganic salts, monomers, etc. Indeed, BZ has been coupled to polymerization reaction^22^ and polymerization-induced self-assembly^23–25^.

It is relatively straightforward in the pH network to map the accept and reject states into the dominant ionized form of malonic acid:

- Accept: the malonates in equal proportion ([C_3_H_3_O_4_^−^] = [C_3_H_2_O_4_^2−^]) dominate while [C_3_H_4_O_4_] is negligible
- Type 1 reject: [C_3_H_4_O_4_] $\ge$ [C_3_H_3_O_4_^−^] dominate while [C_3_H_2_O_4_^2−^] is negligible.
- Type 2 reject: [C_3_H_2_O_4_^2−^] dominates, and [C_3_H_4_O_4_] and [C_3_H_3_O_4_^−^] are negligible.

For BZ, since it involves so many intermediate concentrations this type of mapping from states to chemical species becomes cumbersome, and therefore a mapping to dominant pathway transitions and oscillatory signatures is more convenient^1^.

In connection with the thermodynamic metric and its use, it is not surprising that the same thermodynamic accept/reject criterion that was developed for recognizing a context-sensitive language with the Turing machine can be applied to the Dyck language without any modification. This, of course, was to be expected in view of the generality of the thermodynamic variables used for this criterion. Indeed, our metric brings with it various strengths. For example, the thermodynamic-based metric for the Rule-2 criterion in the BZ-TM facilitates and translates the chemical response into a more intuitive accept/reject criterion for the user. Even without optimizing specifically the aliquot recipes, this criterion provides a nearly constant value for words in the Dyck language. Thus, words in the language accepted by the automaton, are efficient in keeping a balance between the oxidation and reduction pathways even as the word length increases. In contrast, words with excess open parentheses enhance the oxidation pathway more than the reduction pathway, and thus the change in Gibbs free energy is not kept constant and varies with word length for sequences not in the automaton’s accepted language.

Following the same logic, a thermodynamic Rule-2 criterion can also be formulated for the pH-based machine: the enthalpy yield is maximum and independent of word length for Dyck words and below this maximum for rejected strings^1^. However, in this case using the thermodynamic criterion does not offer any clear advantage compared to a purely chemical interpretation.

The pH-based 1-stack PDA used a shorter time interval τ between aliquots^1^, 3 min vs. 7.5 min in the BZ-based TM. (The BZ-based TM requires a longer time interval τ between symbols because of the induction time of the reaction and τ should be longer than the induction time + 2 oscillations which was the criterion we chose in this case^1^). Hence, language recognition was executed faster with the pH-based PDA than with the BZ-based TM. In both types of chemical automata, the aliquot recipes, initial conditions, operating conditions and reactor configuration can be optimized to reduce the time interval and speed up computations. But ultimately the slowest reaction in the mechanism limits the maximum speed for computation. Faster kinetic rates and smaller number of reactions makes the pH-based machine faster than the BZ machine for recognizing the Dyck language. Again, this is in consonance with abstract automata theory, the 1-stack PDA is faster than the Turing Machine for recognizing balanced parentheses so long as we assume that computation time is proportional to the number of state transitions the automaton carries out. The Turing machine with all its changes of direction (right and left) involves more state transitions and reads and overwrites repeated times a symbol cell, whereas a 1-stack PDA reads only once and executes one state transition per symbol. Hence, given a language, say *L*_2_, the performance in computing time and efficiency is different for the two automata, with the faster automaton being the least complex.

We can also compare how the three native chemical automata recognize L_1_. Their distinct chemical signatures for accept and reject states are summarized in Table 2. The reconfiguration was straightforward since the chemical signatures are not only different quantitatively but also qualitatively (e.g. no oscillations vs. oscillation).

**Table 2:** Comparison of the chemical signatures of accept and reject states for the precipitation-based FA, pH-based 1-stack PDA and the BZ-based TM that recognize L_1_.

	Accept	Type 1 Reject (S_1_)	Type 2 Reject (S_2_)
Chemical FA	Onset of precipitate	No precipitate	No precipitate
pH-based 1-stack PDA	ΔpH change sign	ΔpH > 0 or max pH	ΔpH < 0 or min pH
BZ-based TM	Onset of oscillations	(non-oscillatory) high V	(non-oscillatory) low V

## Conclusions

We call a computation the mechanical (i.e. systematic) processing of available input information into some output information which is then used for some appropriate purpose. All computations can be cast as combinations of language recognition problems by suitable computing automata^5^. These automata parallel the Chomsky hierarchy of languages which form an inclusive hierarchy^4^.

Using chemistry, the most complex systems that we know, living systems, have the processing of information at their very core. They can efficiently process information for a variety of tasks which include molecular replication, transcription, translation, regulation, metabolism or epigenetics^26^. Information expressed with chemistry is one of the hallmarks of life. This “information is not a disembodied abstract entity; it is tied to a physical representation”^27^. It is chemical information being processed directly by chemical automata without having to resort to any kind of auxiliary “simulation”^28^. Indeed, living systems outperform supercomputers in their thermodynamic efficiency, as shown in ref. ^29^ for the efficiency of translation in the central dogma of biology.

Purely chemical computation would exclusively use chemical “hardware” and “software”: with input information expressed chemically, its processing taking place via the pathways of suitable chemical reactions and producing an output that is also chemical (in terms of molecular states) or chemically usable, such as the values of suitable chemical state functions for the system of computing chemicals.

In this paper we have shown that native chemical automata can be configured to recognize languages at their level of the Chomsky hierarchy as well as in lower levels, suggesting that chemical reaction networks can also be categorized in an inclusive hierarchy in terms of their computational capabilities. Here we have seen experimentally that the example of the BZ network is an easily reconfigurable and capable network. Known^1^ to recognize L_3_ we have shown how it can be reconfigured to recognize the *Dyck* language (L_2_) or the regular language L_1_, both at lower levels in the Chomsky hierarchy than the level of L_3_. The pH-automaton, known to recognize L_2_, can also be reconfigured to recognize L_1_. However, as expected, the chemical FA based on a simple bimolecular reaction cannot be reconfigured to recognize L_2_ or the context-sensitive language L_3_. Similarly, the pH-based automaton cannot be reconfigured to recognize L_3_. This is because these simpler reaction networks do not have the necessary chemical intermediates or sufficiently distinct dominant pathways. Oscillatory pH-based networks, or other chemical oscillators, whose reaction networks enable at least 4 distinct reactant-dependent dominant pathways can be configured to recognize the L_3_ language since this is the chemical requirement to achieve conformity with the abstract automata description (e.g. congruence between the observed and abstract state transitions).

We have also seen here that the accept/reject criteria for each of the above automata, from the FA to the 1-stack PDA to the Turing machine can be formulated in terms of a thermodynamic metric introduced in ref. ^1^, which we call “Area”. This thermodynamic metric not only simplifies the accept/reject interpretation from evaluating two oscillatory features into a single valued metric, but also enables optimization of the chemical automaton such that the change in Gibbs free energy, for example, is maintained constant for words in the language regardless of their length, and with subsequent implications for the energetics of computation^6^ and the simplicity of design of effective native chemical computing machinery.

New hardware and computer frameworks are needed for carrying out more complex computations, for accelerating computation speed, for parallelizing computations in novel ways, for computing more efficiently in energetic terms or to control and execute the design of new materials using chemistry^22–24^ in ways in which silicon computing cannot compete. All the above features can be improved using native chemical computation, although the specific details and associated strategies for practical applications are still undeveloped.

Indeed, bioinspired and chemical unconventional computing frameworks are thus promising, but to really compete with silicon-based systems they must be reconfigurable, “*scalable and capable*”^30^. Here we have shown how the reconfigurability of native chemical automata is indeed possible. This opens up the many opportunities provided by chemical computing^31^.

## Methods

### Recognition of the Dyck language by Belousov-Zhabotinsky chemistry

#### Materials

Commercially available analytical grade reagents were used without further purification: sodium bromate ${{\rm{NaBrO}}}_{3}$ (Alfa Aesar), malonic acid ${{\rm{CH}}}_{2}{({\rm{COOH}})}_{2}$ (Alfa Aesar), Tris(2,2′-bipyridyl) dichloro ruthenium(II) hexahydrate ${{\rm{Ru}}({\rm{bpy}})}_{3}{{\rm{Cl}}}_{2}(6{{\rm{H}}}_{2}{\rm{O}})$ (Sigma Aldrich) and sulfuric acid solution ${{{\rm{H}}}_{2}{\rm{SO}}}_{4}$ (10 N/5 M, Fisher Chemical). Deionized water (12 Megaohm) was used to prepare the following stock solutions: 2 M ${{\rm{NaBrO}}}_{3}$, 3.5 M ${{\rm{CH}}}_{2}{({\rm{COOH}})}_{2}$ and 0.0125 M ${{\rm{Ru}}({\rm{bpy}})}_{3}{{\rm{Cl}}}_{2}(6{{\rm{H}}}_{2}{\rm{O}})$.

#### Initial conditions

The initial solution was prepared by mixing 34.40 mL of deionized water and 4.8 mL of 5 M sulfuric acid solution giving the following initial concentration: [H^+^] = 0.61 M.

#### Experimental setup

The experiments were carried out in a semibatch reactor under controlled temperature conditions. A 100 mL volume and 50 mm diameter Pyrex® glass beaker with the initial solution was submerged in a 7 L refrigerated circulating bath (VWR MX7LR) at a constant setpoint of 22.0 °C. The reaction mixture was stirred at 400 rpm with a Teflon-coated magnetic stirbar (VWR® Spinbar® Polygon 6.4 × 35 mm) and a submersible magnetic stirrer (2Mag Mixdrive 1 eco and 2Mag Mixcontrol eco). The change in the oxidation-reduction (redox) potential of the reaction mixture was monitored with an electrode system composed of a Pt-working electrode and a mercury sulfate reference electrode (Koslow 5100 A) connected to a benchtop meter (SperScientific). The temperature in the solution was monitored with an RTD sensor probe (Omega PR-13-2-100-1 and signal conditioner RTD SPRTX-S1) and was maintained by the circulating bath at 22.0 ± 0.3 °C during the experiment. Redox and temperature data were recorded with Labview Signal Express at a frequency of 5 data points per second. The refrigerated circulating bath opening was covered with aluminum foil to avoid light interferences since the used catalyst is photosensitive.

#### Alphabet assignment

“(”- An aliquot of the oxidizer, sodium bromate. It affects dominantly the oxidation of catalyst, the autocatalytic production of the key intermediate HBrO_2_, and the bromination of malonic acid. This translates into faster oscillations (smaller period), a reduction of the amplitude of oscillations, and a shift of the oscillation towards higher redox potential values. “)” – An aliquot of the reductant, malonic acid (or other equivalent weak organic acids used in BZ). It affects dominantly the bromination of malonic acid and the reduction of the catalyst. This translates into faster oscillations (smaller period), but the amplitude of oscillations is maintained nearly constant. “#” – An aliquot of the catalyst, in this case tris(2,2′-bipyridyl) dichloro ruthenium(II). If affects simultaneously the oxidation and the reduction of the catalyst, i.e. the core of the reaction mechanism. This translates into a deceleration of the oscillations (larger period), an increase of the amplitude and an overall shift of the oscillations towards higher redox potential values.

#### Symbol recipes

For each open parenthesis in the sequence being checked, 2.0 ± 0.03 mL aliquot of 2.0 M stock sodium bromate solution was pipetted (Eppendorf Research Plus pipette 0.5–5 mL) into the reactor, hence incrementing the bromate concentration in the reactor by 0.10 M. For each closed parenthesis, 0.343 ± 0.004 mL aliquot of 3.5 M stock malonic acid solution was pipetted (Eppendorf Research Plus pipette 0.1–1 mL) into the reactor, hence incrementing the malonic acid concentration in the reactor by 0.03 M. The initial catalyst concentration end-of-expression symbol is implemented as a 0.800 ± 0.006 mL aliquot of 0.0125 M stock ruthenium complex solution (Eppendorf Research Plus pipette 0.1–1 mL) hence increasing the catalyst concentration in the reactor by 0.00025 M.

#### Experimental procedure

The initial solution is kept in the bath until temperature is stabilized. Then, the initial catalyst aliquot (0.800 ± 0.006 mL of 0.0125 M stock ruthenium complex solution) is pipetted. (Note that this is equivalent to introducing the beginning and end of sequence symbol, #, to denote in this case the beginning of sequence.). The sequence checking procedure starts 450 s later by pipetting the first parenthesis in the expression. All subsequent symbols were pipetted after the previous symbol had been processing in the reactor during 450 s. The precision of the additions of the aliquots was within ±2 s. The 450 seconds long interval was selected to give enough time for the chemical system to react and compute the symbol but at the same time minimizing the gas production that could interfere with the redox measurement. The recipes for the initial catalyst concentration, open parenthesis and closed parenthesis were chosen to ensure that the reaction would start to oscillate as soon as both types of parentheses were present in the solution and within the 450 s time interval, and to give measurable changes in the amplitude and frequency of oscillations with the available setup and monitoring system.

#### Data analysis

For each expression, the experiment was run three times. The recorded data were analyzed, visualized and plotted in Matlab® (R2015b v. 8.6.0.267246). Matlab® Signal Processing Toolbox was used to read the amplitude and period from the recorded data for each of the three repetitions of the same expression. The procedure is as follows. First, the peaks of the oscillations are detected, followed by the detection of the troughs. Then, the period is obtained as the time elapsed between two consecutive peaks. The amplitude is defined as the distance between the redox value of the peak minus the redox value of the previous most near trough. Error bars were then estimated as symmetric error bars in the form of: mean ± standard deviation, where the standard deviation was normalized by the number of observations (i.e. 3). Hence, the mean final period ${T}_{\#}$ and the standard deviation of the final period ${std}({T}_{\#})$, the mean final amplitude ${L}_{\#}$, and the standard deviation of the final amplitude *std*(*L*_*#*_,) were estimated respectively as:

$$\begin{array}{ccl}{T}_{{\rm{\#}}} & = & \frac{{\sum }_{i=1}^{3}{T}_{i}}{3}\\ {L}_{{\rm{\#}}} & = & \frac{{\sum }_{i=1}^{3}({V}_{peak,i}-{V}_{trough,i})}{3}\\ std({T}_{{\rm{\#}}}) & = & \sqrt{\frac{{\sum }_{i=1}^{3}{({T}_{i}-{T}_{{\rm{\#}}})}^{2}}{3}}\\ std({L}_{{\rm{\#}}}) & = & \sqrt{\frac{{\sum }_{i=1}^{3}{({L}_{i}-{L}_{{\rm{\#}}})}^{2}}{3}}\end{array}$$

And the error bars for the amplitude ${e}_{L}$, and period ${e}_{T}$, are given respectively by:

$$\begin{array}{c}{e}_{L}={L}_{{\rm{\#}}}\pm std({L}_{{\rm{\#}}})\\ {e}_{T}={T}_{{\rm{\#}}}\pm std({T}_{{\rm{\#}}})\end{array}$$

The area ${A}^{({Word})}$ was integrated approximately via the trapezoidal method with spacing 0.2 s (same as sampling interval in the data) by means of the trapz command in Matlab®. Note that for this integration we have used the last 7 min of the 7.5 min interval after the end-of-expression symbol was added, and 7 min were also used in the first term of the area equation. Again, the error bars for the area are given as the mean value plus/minus one standard deviation: *e*_*A*_ = *A*^(*Word*)^ ± std(A^(*Word*)^).
